# Supplementary material for: A machine-readable specification for genomics assays
Source: bioRxiv. 2023 Jul 18:2023.03.17.533215. Originally published 2023 Mar 21. Preprint. [Version 2] doi: 10.1101/2023.03.17.533215 (PMC10055303; doi:10.1101/2023.03.17.533215)
Supplement: Supplement 1 [file media-1.pdf]

Supplementary Information:
A machine-readable specification for genomics assays

A. Sina Booeshaghi[1*], Xi Chen[2], and Lior Pachter[1,3*]

1. Division of Biology and Biological Engineering, California Institute of Technology, Pasadena, California
2. School of Life Sciences, Southern University of Science and Technology, Shenzhen, China
3. Department of Computing and Mathematical Sciences, California Institute of Technology, Pasadena, California

*Address correspondence to abooesha@caltech.edu & lpachter@caltech.edu

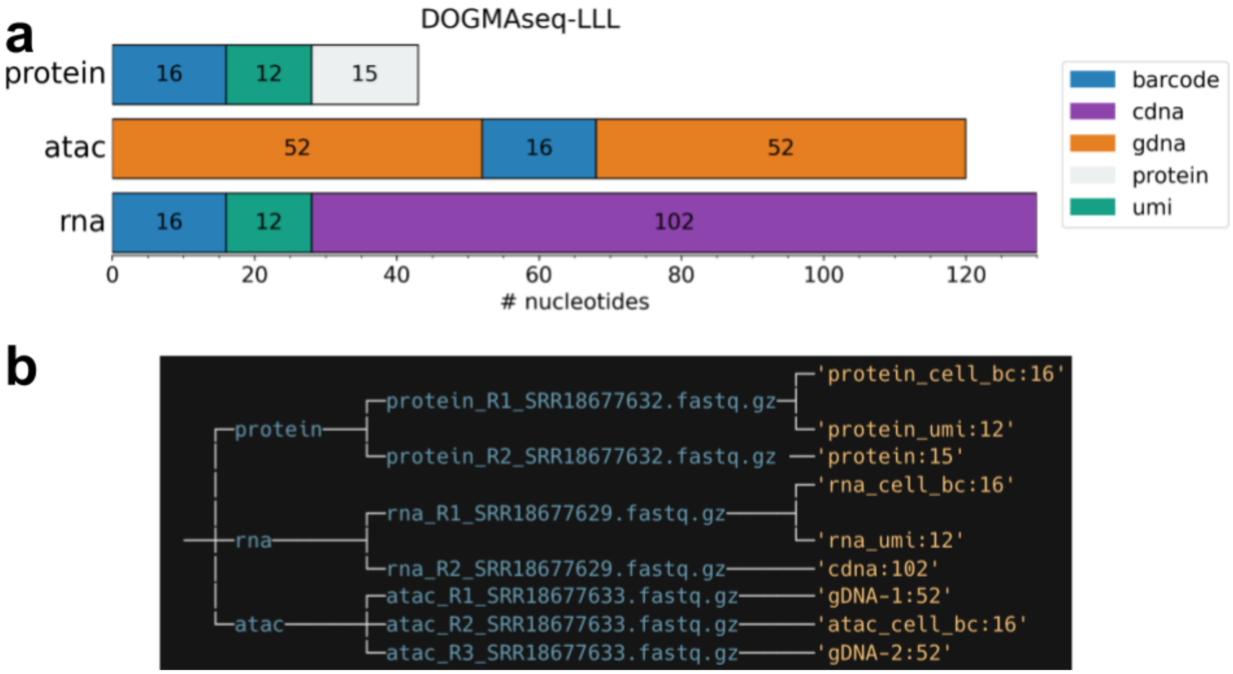

Supplementary Figure 1: *seqspec* read structure of the DOGMAseq-LLL (Xu et al. 2022) assay annotated by (a) atomic regions and their lengths. The (b) ordered-tree representation of the reads.

References

Xu, Zhongli, Elisa Heidrich-O'Hare, Wei Chen, and Richard H. Duerr. 2022. "Comprehensive Benchmarking of CITE-Seq versus DOGMA-Seq Single Cell Multimodal Omics." *Genome Biology* 23 (1): 1–17.
